# Physical comorbidities in men with mood and anxiety disorders: a population-based study

**DOI:** 10.1186/1741-7015-11-110

**Published:** 2013-04-24

**Authors:** Livia Sanna, Amanda L Stuart, Julie A Pasco, Mark A Kotowicz, Michael Berk, Paolo Girardi, Sharon L Brennan, Lana J Williams

**Affiliations:** 1 Unit of Psychiatry, Neurosciences, Mental Health and Sensory Organs Department (NeSMOS), Faculty of Medicine and Psychology, Sant’Andrea Hospital, Sapienza University of Rome, Via di Grottarossa 1035–1037 00189, Rome, Italy; 2 School of Medicine, Deakin University: The Geelong Hospital, PO Box 281, Geelong 3220, Australia; 3 Department of Psychiatry, The University of Melbourne, Parkville 3010, Australia; 4 NorthWest Academic Centre, Department of Medicine, The University of Melbourne, Western Health, 176 Furlong Road, St Albans 3021, Australia; 5 Australian Institute of Musculo-Skeletal Science, NorthWest Academic Centre, The University of Melbourne, 176 Furlong Road, St Albans 3021, Australia; 6 Orygen Youth Health, 35 Poplar Road, Parkville 3052, Australia; 7 Florey Institute for Neuroscience and Mental Health, The University of Melbourne, Parkville 3010, Australia

**Keywords:** Mood disorder, Anxiety disorder, Comorbidity, Medical burden, Population-based study, Physical illness

## Abstract

**Background:**

The mind-body nexus has been a topic of growing interest. Further data are however required to understand the specific relationship between mood and anxiety disorders and individual physical health conditions, and to verify whether these psychiatric disorders are linked to overall medical burden.

**Methods:**

This study examined data collected from 942 men, 20 to 97 years old, participating in the Geelong Osteoporosis Study. A lifetime history of mood and anxiety disorders was identified using the Structured Clinical Interview for DSM-IV-TR Research Version, Non-patient edition (SCID-I/NP). The presence of medical conditions (lifetime) was self-reported and confirmed by medical records, medication use or clinical data. Anthropometric measurements and socioeconomic status (SES) were determined and information on medication use and lifestyle was obtained via questionnaire. Logistic regression models were used to test the associations.

**Results:**

After adjustment for age, socioeconomic status, and health risk factors (body mass index, physical activity and smoking), mood disorders were associated with gastro oesophageal reflux disease (GORD), recurrent headaches, blackouts and/or epilepsy, liver disorders and pulmonary disease in older people, whilst anxiety disorders were significantly associated with thyroid, GORD and other gastrointestinal disorders, and psoriasis. Increased odds of high medical burden were associated with both mood and anxiety disorders.

**Conclusions:**

Our study provides further population-based evidence supporting the link between mental and physical illness in men. Understanding these associations is not only necessary for individual management, but also to inform the delivery of health promotion messages and health care.

## Background

For centuries, it has been hypothesised and debated that a mind-body interaction exists. Over time, an evidence base has grown to support this notion, yet an incomplete understanding prevails [1,2]. Rates of depression and anxiety are generally higher among the physically ill than in the general population; however, results vary according to the type and severity of the chronic disease and methods of ascertainment [3,4].

Within clinical samples, patients with depression have been found to have a significantly higher number of somatic symptoms [5] and a higher risk of chronic diseases [6-8], such as coronary heart disease [9,10], congestive heart failure [11], stroke and dementia [12], diabetes [13,14], gastro oesophageal reflux disease (GORD) [15], osteoarthritis and rheumatoid arthritis [16], psoriasis [17], cancer [18], neurological disorders [19], pulmonary disease [20], liver disease [21], thyroid disorders [22] and asthma [23,24], as well as non-specific syndromes such as obesity [25], anaemia [26], renal dysfunction [27], chronic fatigue syndrome [28], chronic headaches [24], and chronic pain [29]. The strength of the association as well as the extent of the evidence varies for each medical condition.

Similarly, anxiety disxorders, have been shown to be related to several medical conditions, such as cardiovascular disease [30], cancer [18], obesity [31] and other metabolic disorders [32], irritable bowel syndrome [33], gastrointestinal problems [34], GORD [15], thyroid disorders [35], psoriasis [17] and a higher number of medical symptoms [36]. In addition, as recently reviewed by Culpepper and colleagues, migraine and chronic pain, diabetes, peptic ulcer, arthritis and pulmonary disease are also associated [2].

The co-occurrence of mood or anxiety disorders and physical illness worsens the impact of symptom burden [36], impacts disease course, links to the deteriorating patient’s health status and functioning [7,37], affects medication response and treatment adherence [38], and increases the risk of complications [39] and even death [37]. Moreover, the comorbidity between mental and physical illness is relevant in terms of role impairment and work performance [24], as well as quality of life and health service use and costs [40].

The causal pathway between comorbid mental and physical illness is complex and remains unclear. It has been suggested that the bi-directionality of this relationship may involve several mechanisms [41,42] including biological (for example, increased pro-inflammatory cytokines, hypothalamic-pituitary axis deregulation, autonomic nervous system dysfunction, serotonin depletion, metabolic, immune and endocrine changes) [43], psychological (for example, behavioural, psychodynamic and cognitive factors) [44] and social (for example, impaired social support, loneliness, and social disengagement) [45]. Furthermore, psychotropic medication use has been linked to a higher incidence of an array of health outcomes, including diabetes, falls and bleeding, osteoporosis, and sudden cardiac death [46-48].

Enhancing the quality of care for those with mental illness not only can improve mood and anxiety symptoms, but also physical health in patients with comorbid mental and physical illness [49]. Furthermore, treatment for these disorders may influence each other [50]. Thus, estimating the prevalence and understanding the association between mood and anxiety disorders and physical illness is important in order to make more accurate diagnoses and to provide integrated and effective treatment, with regard to syndrome reciprocal influences and medication interactions. Given these data, we aimed to describe the relationship between comorbid mental and physical illness in a large, representative sample of men residing in Australia, utilising structured clinical interviews, medical records, and clinical and self-reported data collected as part of the Geelong Osteoporosis Study (GOS) [51].

## Methods

### Participants

Data were derived from the GOS male cohort, a study originally designed to investigate the epidemiology of osteoporosis in Australia. Originally, an age-stratified, population-based sample of adult men (n = 1,540; response 67%, 20 to 97 years old) was randomly-selected from the Australian Electoral Commission (AEC) rolls for the Barwon Statistical Division between 2001 and 2006. The Barwon Statistical Division is situated in South-Eastern Australia, comprising both rural and urban communities. Further details of the study have been published elsewhere [51].

Of those invited to participate in the five-year follow-up study (n = 1,540), 141 had died, 41 had left the study region, 16 were unable to provide written informed consent, 139 were not able to be contacted and 225 declined to participate resulting in 978 participants (81% of the eligible baseline sample). For this analyses, those who did not undergo psychiatric assessment (n = 17) or provide medical information (n = 19) were excluded, resulting in a final sample of 942 eligible men. This study was approved by the Barwon Health Human Research Ethics Committee and written informed consent was obtained from all participants.

### Assessments

#### Psychiatric measures

The Structured Clinical Interview for the Diagnostic and Statistical Manual of Mental Disorders, 4th edition, text revision (DSM-IV-TR) Research Version, Non-patient edition (SCID-I/NP) [52] was used to identify those who had ever experienced a mood disorder, including major depressive disorder, bipolar disorder (types I and II), dysthymia, minor depression, mood disorder due to general medical condition (GMC) and substance-induced mood disorder; and/or an anxiety disorder including panic disorder, agoraphobia, social phobia, specific phobia, obsessive compulsive disorder, generalised anxiety disorder, anxiety disorder due to a medical condition, substance-induced anxiety disorder or anxiety disorder not otherwise specified. All interviews were conducted by personnel with qualifications in psychology, who were trained using live and videotaped interviews under the supervision of a psychiatrist.

#### Clinical measures

Height was measured to the nearest 0.1 cm using a wall-mounted stadiometer and body weight was measured to the nearest 0.1 kg using electronic scales. Body mass index (BMI; kg/m^2^) was calculated from these measurements.

Lumbar spine and femoral neck bone mineral density (BMD) was measured by dual energy X-ray absorptiometry (DXA) using GE Lunar Prodigy (Prodigy GE Lunar Madison, WI, USA). Long term stability of the machine was tested three times weekly by scanning an anthropomorphic phantom (Hologic). Low bone mass was classified according to the World Health Organisation definition; one or more standard deviations below the young normal mean at the femoral neck or spine (L2-L4, posterior-anterior projection) [53].

#### Medical conditions

The presence of medical conditions (lifetime) was self-reported and confirmed by medical records, medication use or clinical data, where possible. Musculoskeletal disease was defined by low BMD or self-reported osteoarthritis, ankylosing spondylitis, psoriatic arthritis, rheumatoid arthritis, muscle disease or weakness, low trauma fracture, or self-reported or medicated gout (agents used in gout or hyperuricaemia). Thyroid disorders included self-reported or medicated (thyroid hormones and anti-thyroid agents) hyperthyroidism, hypothyroidism, Graves’ disease, Hashimoto’s disease, thyroiditis and other unspecified thyroid conditions. Metabolic risk factors included a diagnosis of diabetes, defined by self-report or current medication use (oral hypoglycaemic agents and insulin preparations), obesity defined by a BMI >30 kg/m^2^[54], self-reported or medicated hypercholesterolemia (use of cholesterol-lowering agents) and hypertension. Hypertension was characterised by a combination of self-report and current medication use (antihypertensive, beta-adrenergic blocking agents and diuretics) or a systolic blood pressure of >140 mmHg or diastolic blood pressure of >90 mmHg. Diagnosis of GORD was based on self-report as were gastrointestinal diseases which include peptic ulcer, chronic gastritis and causes of malabsorption, such as pancreatitis, gastric surgery, chronic diarrhoea, irritable bowel syndrome, inflammatory bowel syndrome and coeliac disease. Cardiovascular disease was defined by a diagnosis of hypertension (as detailed above) or a combination of self-reported or medicated (beta-adrenergic blocking agents, diuretics, anti-arrhythmic, anti-angina, cardiac inotropic agents, adrenergic stimulants, other cardiovascular agents) stroke, transient ischaemic accident (TIA), ischaemic cardiomyopathy, arrhythmia and valve and vascular diseases. Syncope and seizures included self-reported epilepsy, blackouts, fainting and dizzy spells. Self-reported migraine and other recurrent headaches were grouped together. Lifetime pulmonary disease included self-reported asthma, emphysema and chronic bronchitis. Psoriasis was defined by self-report. Liver disease diagnosis, including fatty liver, hepatitis, cirrhosis and liver failure, was also based on self-report. Cancer was defined as any malignant tumour including non-melanoma skin cancer. High medical burden was classified as a diagnosis of three or more conditions listed above.

#### Other measures

Smoking status, medication use and physical activity level were obtained by self-report [51]. Reported medication use was cross-referenced with the participants’ list of medications or containers and was considered current if the participants reported use at the time of assessment. Men were classified as active if they participated in light to vigorous activity on a regular basis. Alcohol consumption (average grams consumed daily over a 12 month period) was determined by a validated food frequency questionnaire [55]. Tobacco smoking was documented and grouped as current or not. In order to determine socioeconomic status (SES), residential addresses were matched to the corresponding Australian Bureau of Statistics (ABS) Census Collection District (CCD). ABS software was used to determine the Socio-Economic Index for Areas (SEIFA) from the 2006 ABS Census data. SEIFA values summarize the characteristics of subjects within an area, thereby providing a single measure to rank the level of disadvantage at the area level, not of the individual subject. SEIFA values indicate the level of advantage or disadvantage within each CCD, small geographic areas that incorporate approximately 250 houses [56]. These values inform the Index of Relative Socioeconomic Advantage/Disadvantage (IRSAD), which accounts for various socioeconomic parameters including high and low income, educational attainment ranging from no qualifications to a tertiary degree or higher, the type of occupation from unskilled employment to professional positions, and some measure of wealth (such as owning a car, or number of bedrooms in a dwelling). The relative SES of participants was ascertained by categorizing the sample into quintiles based on cutpoints for the study region [57-59].

### Statistical analysis

Differences in characteristics between those with and without mood and anxiety disorders were compared using chi-square analyses for discrete variables and Mann–Whitney for non-parametric continuous variables. Logistic regression was used to calculate odds ratios (OR) with 95% confidence interval (95% CI) of lifetime medical conditions for those with lifetime mood/anxiety disorders in comparison to those with no mood/anxiety disorder. Models were adjusted for age (model I) and then for age, BMI, SES, physical activity, alcohol consumption and smoking status (model II). Interactions between exposure variables were examined. Each analysis was performed with and without participants with mood disorders due to GMC; no differences in outcome were detected, therefore, those with mood disorder due to GMC were included in all analyses. Values of *P* <0.05 were accepted as significant (including interaction terms). Statistical analyses were performed using Minitab (version 16; Minitab, State College, PA, USA).

## Results

A total of 160 men (17%) had a lifetime history of mood disorder, of which 139 (86.9%) were identified with major depressive disorder, 10 (6.3%) dysthymia, 9 (5.6%) minor depression, 3 (1.9%) bipolar disorder, 3 (1.9%) mood disorder due to a GMC and 2 (1.3%) substance induced mood disorder. Of the 60 participants (12.2%) with a history of anxiety disorder, 23 (38.3%) met criteria for panic disorder, 15 (25%) post-traumatic stress disorder, 7 (11.7%) specific phobia, 6 (10%) social phobia, 5 (8.3%) generalized anxiety disorder, 5 (8.3%) obsessive compulsive disorder and 3 (5%) anxiety disorder not otherwise specified. Participants with a history of mood or anxiety disorder were younger than those without; otherwise the groups were similar (Table 1).

**Table 1:** Characteristics for the total group and men with or without lifetime history of mood disorder and anxiety disorder

			**Mood disorder**			**Anxiety disorder**		
		**Total**	**Yes**	**No**	***P***	**Yes**	**No**	***P***
		**Number = 942**	**Number = 160**	**Number = 782**		**Number = 60**	**Number = 882**	
Age at interview (years)		59.3 (45.7, 73.2)	52.8 (42.6, 64.4)	60.9 (46.9, 75.4)	<0.001	56.2 (41.1, 64.3)	59.9 (46.0, 74.0)	0.013
Body mass index (kg/m^2^)		27.1 (24.7, 29.6)	27.1 (24.6, 29.6)	27.1 (24.8, 29.8)	0.671	27.7 (25.1, 30.3)	27.1 (24.6, 29.6)	0.257
Socioeconomic status (IRSAD)	Quintile 1 (most disadvantaged)	153 (16.2%)	24 (15.0%)	129 (16.5%)	0.581	6 (10.0%)	147 (16.7%)	0.147
	Quintile 2	189 (20.1%)	27 (16.9%)	162 (20.7%)		12 (20.0%)	177 (20.1%)	
	Quintile 3	182 (19.3%)	29 (18.1%)	153 (19.6%)		8 (13.3%)	174 (19.7%)	
	Quintile 4	202 (21.4%)	37 (23.1%)	165 (21.1%)		13 (21.7%)	189 (21.4%)	
	Quintile 5	216 (22.9%)	43 (26.9%)	173 (22.1%)		21 (35.0%)	195 (22.1%)	
Physically active		674 (71.9%)	108 (67.5%)	566 (72.8%)	0.179	46 (76.7%)	628 (71.5%)	0.392
Smokers (current)		105 (11.2%)	23 (14.5%)	82 (10.5%)	0.153	10 (16.7%)	95 (10.8%)	0.166
Alcohol consumption (g/day)		12.4 (2.2, 28.8)	17.3 (2.0, 34.3)	11.9 (2.2, 28.3)	0.156	20.3 (2.8, 28.7)	12.0 (2.8, 28.7)	0.179
Musculoskeletal disease		706 (77.0%)	123 (79.4%)	583 (76.5%)	0.443	47 (79.7%)	659 (76.8%)	0.614
Thyroid disorders		21 (2.2%)	4 (2.5%)	17 (2.2%)	0.799	4 (6.7%)	17 (1.9%)	0.016
Metabolic risk factors		665 (74.3%)	107 (72.8%)	558 (74.6%)	0.646	46 (79.3%)	619 (74.0%)	0.367
GORD		124 (13.2%)	29 (18.1%)	95 (12.1%)	0.042	16 (26.7%)	108 (12.2%)	0.001
Gastrointestinal disorders		164 (17.4%)	31 (19.4%)	133 (17.0%)	0.472	16 (26.7%)	148 (16.8%)	0.051
Recurrent headaches		50 (5.3%)	15 (9.4%)	35 (4.5%)	0.012	2 (3.3%)	48 (5.4%)	0.481
Syncope and seizures		157 (16.7%)	37 (23.1%)	120 (15.4%)	0.016	12 (20.0%)	145 (16.4%)	0.474
Cardiovascular disease		598 (68.5%)	91 (65.5%)	507 (69.1%)	0.401	34 (63.0%)	564 (68.9%)	0.366
Pulmonary disease	All	178 (18.9%)	-	-		11 (18.3%)	167 (18.9%)	0.908
	<60 years old	90/478 (18.8%)	19/109 (17.4%)	71/369 (19.2%)	0.671	-	-	-
	≥60 years old	88/464 (19.0%)	18/51 (35.3%)	70/416 (17%)	0.002	-	-	-
Liver disorders		55 (5.8%)	16 (10%)	39 (5%)	0.014	3 (5.0%)	52 (5.9%)	0.775
Cancer		162 (17.2%)	20 (12.5%)	142 (18.2%)	0.084	8 (13.3%)	154 (17.5%)	0.412
Psoriasis		41 (4.3%)	9 (5.6%)	32 (4.1%)	0.387	5 (8.3%)	36 (4.1%)	0.118
High medical burden		548 (62.2%)	90 (62.9%)	458 (62.1%)	0.843	40 (70.2%)	508 (61.7%)	0.199

Values are given as median (interquartile range) or number (%). Missing data: BMI, number = 28; physically active, number = 4; smokers, number = 5; musculoskeletal disease, number = 25; metabolic risk factors, number = 47; cardiovascular disease, number = 69; high medical burden, number = 61. GORD, gastro oesophageal reflux disease; IRSAD, Index of Relative Socioeconomic Advantage/Disadvantage.

### Mood disorder

After adjustments for age, mood disorder was associated with increased odds of GORD (OR 2.41, 95% CI 1.48 to 3.00, *P* = 0.027), recurrent headaches (OR 2.46, 95% CI 1.29 to 4.69, P = 0.007), syncope and seizures (OR 2.41, 95% CI 1.31 to 3.09, *P* = 0.002) and liver disorders (OR 2.41, 95% CI 1.40 to 4.98, *P* = 0.003). Furthermore, a history of mood disorder was associated with increased odds of high medical burden (age-adjusted OR 1.80, 95% CI 1.16 to 2.77, *P* = 0.008). Mood disorders tended to be associated with musculoskeletal disease (OR 1.43, 95% CI 0.93 to 2.21, *P* = 0.104) and gastrointestinal disorders (OR 1.47, 95% CI 0.93 to 2.30, *P* = 0.096). Age was an effect modifier in the association between mood disorders and pulmonary disease. Among older men (≥60 years), a lifetime history of mood disorder was associated with increased odds of pulmonary disease (age-adjusted OR 2.75, 95% CI 1.45 to 5.21, *P* = 0.002); no association was detected for those younger than 60 years (*P* >0.05). These relationships remained significant after further adjustment for BMI, SES, physical activity, alcohol consumption and smoking status (Table 2). No association was observed between mood disorders and thyroid disorders, metabolic risk factors, cardiovascular disease, cancer and psoriasis.

**Table 2:** Age-adjusted (model I) and fully-adjusted (model II) odds ratios for medical comorbidities in men with lifetime history of mood and anxiety disorders

			**Mood disorders**			**Anxiety disorders**		
		**Model**	**Odds ratio**	**95% CI**	***P *value**	**Odds ratio**	**95% CI**	***P *value**
Musculoskeletal disease		I	1.43	(0.93 to 2.21)	0.104	1.39	(0.72 to 2.71)	0.328
		II	1.47	(0.94 to 2.31)	0.092	1.70	(0.85 to 3.43)	0.135
Thyroid disorders		I	1.64	(0.52 to 5.12)	0.396	5.29	(1.63 to 7.14)	0.005
		II	1.81	(0.56 to 5.83)	0.321	5.54	(1.64 to 18.64)	0.006
Metabolic risk factors		I	1.30	(0.85 to 1.99)	0.234	1.92	(0.96 to 3.83)	0.066
		II^a^	1.29	(0.83 to 2.00)	0.265	2.20	(1.07 to 4.53)	0.032
GORD		I	2.41	(1.48 to 3.00)	<0.001	3.84	(2.01 to 7.33)	<0.001
		II	2.67	(1.60 to 4.47)	<0.001	4.25	(2.18 to 8.26)	<0.001
Gastrointestinal disorders		I	1.47	(0.93 to 2.30)	0.096	2.22	(1.20 to 4.10)	0.011
		II	1.46	(0.91 to 2.34)	0.112	2.40	(1.28 to 4.50)	0.006
Recurrent headaches		I	2.46	(1.29 to 4.69)	0.007	0.63	(0.15 to 2.69)	0.537
		II	2.53	(1.30 to 4.96)	0.007	0.67	(0.16 to 2.89)	0.592
Syncope and seizures		I	2.01	(1.31 to 3.09)	0.002	1.46	(0.75 to 2.85)	0.263
		II	2.01	(1.28 to 3.16)	0.002	1.55	(0.78 to 3.07)	0.207
Cardiovascular disease		I	1.27	(0.82 to 1.93)	0.292	1.93	(0.60 to 2.13)	0.708
		II	1.28	(0.82 to 2.01)	0.276	1.15	(0.59 to 2.25)	0.678
Pulmonary disease	All	I	-	-	-	0.95	(0.48 to 1.86)	0.874
		II	-	-	-	1.02	(0.51 to 2.03)	0.956
	<60 years old	I	0.90	(0.52 to 1.58)	0.725	-	-	-
		II	1.01	(0.57 to 1.79)	0.971	-	-	-
	≥60 years old	I	2.75	(1.45 to 5.21)	0.002	-	-	-
		II	2.65	(1.34 to 5.21)	0.005	-	-	-
Liver disorders		I	2.64	(1.40 to 4.98)	0.003	0.97	(0.29 to 3.24)	0.964
		II	3.03	(1.57 to 5.83)	0.001	1.03	(0.30 to 3.46)	0.967
Cancer		I	0.95	(0.56 to 1.61)	0.839	1.03	(0.47 to 2.29)	0.933
		II	0.90	(0.52 to 1.56)	0.706	1.03	(0.46 to 2.32)	0.938
Psoriasis		I	1.63	(0.75 to 3.55)	0.219	2.44	(0.91 to 6.57)	0.076
		II	1.41	(0.51 to 2.91)	0.648	2.77	(1.00 to 7.67)	0.049
High medical burden		I	1.80	(1.16 to 2.77)	0.008	2.82	(1.43 to 5.53)	0.003
		II^a^	1.86	(1.19 to 2.91)	0.007	3.23	(1.61 to6.47)	0.001

^a^Model not adjusted for BMI as it is recorded in the medical condition. Model I – Age-adjusted; Model II – Fully-adjusted including: age, BMI, SES, physical activity, smoking and alcohol consumption. BMI, body mass index; CI, confidence interval; GORD, gastro oesaphogeal reflux disease; SES, socioeconomic status.

### Anxiety disorder

After adjustment for age, anxiety disorder was associated with increased likelihood of thyroid disease (OR 5.29, 95% CI 1.63 to 7.14, *P* = 0.005), GORD (OR 3.84, 95% CI 2.01 to 7.33, *P* <0.001), gastrointestinal disease (OR 2.22, 95% CI 1.20 to 4.10, *P* = 0.011) and high medical burden (OR 2.82, 95% CI 1.43 to 5.53, *P* = 0.003). Anxiety disorder also tended to be associated with metabolic risk factors (OR 1.92, 95% CI 0.96 to 3.83, *P* = 0.066). Further adjustment for BMI, SES, physical activity, alcohol consumption and smoking status did not affect these outcomes (Table 2). The relationship between lifetime anxiety disorder and metabolic risk factors and lifetime anxiety disorders and psoriasis reached significance following adjustment for age, BMI, SES, physical activity, alcohol consumption and smoking status (OR 2.20, 95% CI 1.07 to 4.53, *P* = 0.032 and OR 2.77, 95% CI 1.00 to 7.67, *P* = 0.049, respectively). There were no associations detected between anxiety disorders and musculoskeletal disease, recurrent headaches, syncope and seizures, cardiovascular disease, pulmonary disease, liver disorders or cancer.

## Discussion

Our population-based study reports associations between mood and anxiety disorders and physical illness. Mood disorders were associated with increased risk of many of the disease groups (GORD, neurological features, such as recurrent headaches and syncope and seizures, liver disorders and pulmonary diseases in older men). Anxiety disorders presented a different profile; thyroid disorders, GORD, gastrointestinal disease, metabolic risk factors, and psoriasis were more common among individuals with anxiety disorder. Importantly, mood and anxiety disorders were both associated with high medical burden.

Our results confirm other population-based data investigating the association between mood disorder and GORD [60,61]. In a sample of over 60,000 participants residing in Norway, those identified with depressive, anxiety and comorbid symptoms, as measured by the Hospital Anxiety and Depression Scale, had a two- to three-fold increased risk of reflux symptoms, self-reported severe symptoms of recurrent heartburn or acid regurgitation [62]. Moreover, mood disorders have also been shown consistently to be more prevalent in patients with GORD in clinical practice [15,63].

Chronic headaches are high prevalence disorders, with as many as 46% of the adult population reporting headaches, 11% migraines and 42% tension headaches [64]. Similar to our findings and other population-based studies [65,66], Scott *et al*. [24] utilising data pooled from 18 general population surveys reported those identified with either non-comorbid depression or comorbid depression and anxiety, as measured by the Composite International Diagnostic Interview, had an age and gender adjusted odds ratio of 2.5 and 4.0, respectively, for chronic headaches.

In regard to syncope and seizures, our results support Morgan *et al*. [67] who reported fainting, blackouts and dizziness, for which there is no adequate physical explanation, were associated with undiagnosed psychiatric disorders. Depression has also been shown to co-occur with epilepsy. Recently, the association has been viewed according to the diathesis-stress model, with depression resulting from the chronic stress associated with the threat of recurring seizures, as there is little evidence linking specific epilepsy related factors (for example, focus site) to low mood [68].

Akin to our results, Wilhelm and colleagues [69] reported an association between depression and liver pathology in a population-based survey of 10,641 adults; however, the relationship was not sustained following adjustment for demographic and behavioural confounders including drinking behaviour. Excessive alcohol consumption is known to be highly correlated with liver disease [21]; however, the association persisted following correction for alcohol in our study.

In our sample, only older men with a mood disorder were at increased risk of pulmonary disease. Although the association between asthma and mood disorder is evident across the full adult age range [23], chronic bronchitis and emphysema are more common among older people and most likely contribute to the different pattern observed for older and younger men. Furthermore, pulmonary disease can be considered in stages reflecting severity, a factor we could not explore [70].

Musculoskeletal and gastrointestinal disorders tended to be associated with mood disorders, although we speculate that the heterogeneous groupings we employed may have diluted associations in our sample population. Mood disorders and symptomology have previously been shown to be associated with low BMD and subsequent fracture, where both biological and medication related processes are thought to play a role [71]. Similarly, mood disorders have been shown to be associated with gastrointestinal disorders in both clinical and population-based samples [72-74].

In contrast to other population-based studies, we did not detect a relationship between mood disorders and thyroid disorders, metabolic risk factors, cardiovascular disease, cancer and psoriasis in this sample of men. The link between mood disorders and cardiovascular disease, thyroid disorders and metabolic risk factors, in particular, is well documented [22,24,30]. We hypothesize that these results may be influenced by a healthy participant bias, a common issue where study participants are required to be healthy enough to attend the research centre. Furthermore, disease grouping and data collection limited our ability to identify the degree of illness severity (that is, severe stroke, TIA and medically controlled hypertension were grouped) or time since onset of the physical condition.

As with mood disorders, GORD and gastrointestinal disorders were also associated with anxiety disorders. Anxiety has been previously considered to be a non-disease related factor that impacts negatively on quality of life associated with GORD [75] and, as previously discussed, is commonly observed within population-based samples reporting GORD symptoms [62]. Furthermore, data from the present study concur with previous studies indicating that those with anxiety disorder have higher rates of gastrointestinal disorders [34].

Psoriasis has been previously associated with anxiety disorders. Harter and colleagues [34] demonstrated that patients with a lifetime history of generalised anxiety disorder or panic disorder reported significantly higher lifetime prevalence rates for dermatological disorders, including psoriasis. Why this association is seen with anxiety disorders only in our sample population is unclear.

Thyroid disorders and metabolic risk factors have also been associated with anxiety disorders. In our study, anxiety was linked to a combination of the most common risk factors for metabolic disorders, such as hypertension, diabetes, hypercholesterolemia and obesity, which Culpepper and colleagues reported to be all likely due to a prolonged stress response in patients with untreated anxiety disorder [2]. Similarly, Simon *et al*. [35] reported an increased risk of thyroid disorders in patients with generalised anxiety disorder, social phobia or panic disorder and recommended screening for thyroid dysfunction in patients with anxiety disorders.

Our data suggest that both mood and anxiety disorders are associated with overall medical burden. These findings are consistent with previous studies showing both depression and anxiety to be associated with an increased number of somatic conditions [5,76]. It is plausible that this relationship may be due to mood and anxiety disorders causing physiological changes, as well as poorer self-care and treatment adherence, which in turn increase medical burden [36]. On the other hand, an increased physical burden, causing functional impairment, may result in the development of an anxiety and/or mood disorder, which have been shown to have a negative impact on clinical outcomes [77].

A major strength of our study is that the sample population spans the full adult age range, in contrast to previous studies that have mainly focused on older patients. An age interaction was only evident when exploring the association between mood disorders and pulmonary disease; thus the relationship between mental illness and each of the physical conditions was consistent across all ages. Further strengths of the study include the measurement of mood and anxiety disorders, diagnoses were made utilising semi-structured clinical interviews (SCID-IV), a gold standard tool, and the consideration of several possible confounding factors. However, our observations must be considered with caution. Ascertaining medical conditions by a self-report checklist may be compromised by imperfect recall and response bias error. Although many of these disorders were based on self-report of a physician’s diagnosis, which have been demonstrated to generally agree with medical record data [78], where possible this was confirmed by medical records, medication use or clinical data. The cross-sectional nature of the study does not allow verification of a cause-effect relationship between mental and physical illness, longitudinal studies are needed to determine the directionality of this relationship. Finally, we were unable to make a distinction between diseases with an episodic course and those with ongoing symptoms, which may impact differently on psychological status.

## Conclusion

Our study provides further population-based evidence supporting the link between mental and physical illness in men. Recognising the link between these illnesses is important in order to manage the development of mood and anxiety disorders in response to a medical condition, or the presence of physical pathologies due to predisposing psychiatric disorders. Integrating treatment approaches and monitoring comorbidity of mental and physical illnesses is likely to improve disease course and outcome as well as enhance patients’ functional and health status, potentially reducing healthcare utilisation.

## Abbreviations

ABS: Australian Bureau of Statistics; BMD: Bone mineral density; BMI: Body mass index; CI: Confidence interval; DSM-IV-TR: Diagnostic and Statistical Manual for Mental Disorders, 4th edition, text revision; DXA: Dual energy X-ray absorptiometry; GMC: General medical condition; GORD: Gastro oesophageal reflux disease; GOS: Geelong Osteoporosis Study; IRSAD: Index of Relative Socioeconomic Advantage/Disadvantage; SCID-I/NP: Structured clinical interview for DSM-IV-TR research version, non-patient edition; SEIFA: Socioeconomic Index for Areas; SES: Socioeconomic status; TIA: Transient ischaemic accident.

## Competing interests

Livia Sanna, Amanda L Stuart, Mark A Kotowicz, and Sharon L Brennan have no conflicts of interest, including specific financial interests and relationships and affiliations relevant to the subject matter or materials discussed in the manuscript. Paolo Girardi has in the past three years received research support from Lilly and Janssen, participated in advisory boards for Lilly, Organon, Pfizer, and Schering, and received honoraria from Lilly and Organon. Julie A Pasco has received speaker fees from Amgen, Eli Lilly and Sanofi-Aventis and funding from the Geelong Region Medical Research Foundation, Barwon Health, Perpetual Trustees, the Dairy Research and Development Corporation, The University of Melbourne, the Ronald Geoffrey Arnott Foundation, ANZ Charitable Trust, the American Society for Bone and Mineral Research, Amgen (Europe) GmBH and the NHMRC. Michael Berk has received Grant/Research Support from the NIH, Simons Foundation, CRC for Mental Health, Stanley Medical Research Institute, MBF, NHMRC, Beyond Blue, Geelong Medical Research Foundation, Bristol Myers Squibb, Eli Lilly, Glaxo SmithKline, Organon, Novartis, Mayne Pharma, Servier and Astra Zeneca. He has been a paid consultant for Astra Zeneca, Bristol Myers Squibb, Eli Lilly, Glaxo SmithKline, Janssen Cilag, Lundbeck and Pfizer and a paid speaker for Astra Zeneca, Bristol Myers Squibb, Eli Lilly, Glaxo SmithKline, Janssen Cilag, Lundbeck, Organon, Pfizer, Sanofi Synthelabo, Solvay and Wyeth. Lana J Williams has received Grant/Research support from Eli Lilly, Pfizer, The University of Melbourne, Deakin University and the NHMRC.

## Authors’ contributions

LS and ALS took part in the conception and design of the study, acquisition of the data, data cleaning and statistical analysis, interpretation of the analysis and took primary responsibility for writing the manuscript. JAP and MAK took part in the conception and design of the study, interpretation of the analysis and critically revised the manuscript. MB, PG and SLB took part in the interpretation of data and critically revised the manuscript. LJW took part in the conception and design of the study, interpretation of the analysis, critically revised and supervised the writing of the manuscript. All authors read and approved the final manuscript.

## Pre-publication history

The pre-publication history for this paper can be accessed here:

http://www.biomedcentral.com/1741-7015/11/110/prepub
